# Single cell-derived spheroids capture the self-renewing subpopulations of metastatic ovarian cancer

**DOI:** 10.1038/s41418-021-00878-w

**Published:** 2021-11-29

**Authors:** Tania Velletri, Carlo Emanuele Villa, Domenica Cilli, Bianca Barzaghi, Pietro Lo Riso, Michela Lupia, Raffaele Luongo, Alejandro López-Tobón, Marco De Simone, Raoul J. P. Bonnal, Luca Marelli, Stefano Piccolo, Nicoletta Colombo, Massimiliano Pagani, Ugo Cavallaro, Saverio Minucci, Giuseppe Testa

**Affiliations:** 1 Department of Experimental Oncology, European Institute of Oncology IRCCS, Via Adamello 16, 20139 Milan, Italy; 2 Unit of Gynecological Oncology Research, European Institute of Oncology IRCCS, Via Adamello 16, 20139 Milan, Italy; 3 Department of Oncology and Hemato-Oncology, University of Milan, Via Santa Sofia 9, 20122 Milan, Italy; 4 National Institute of Molecular Genetic (INGM), “Romeo and Enrica Invernizzi”, 20122 Milan, Italy; 5 IFOM, the FIRC Institute of Molecular Oncology, 20139 Milan, Italy; 6 Department of Molecular Medicine (DMM), University of Padua School of Medicine, 35128 Padua, Italy; 7 Division of Gynecologic Oncology, European Institute of Oncology IRCCS, Via Ripamonti 435, 20141 Milan, Italy; 8 Department of Biosciences, University of Milan, Via Santa Sofia 9, 20122 Milan, Italy; 9 Present Address: Cogentech Società Benefit Srl, Parco Scientifico e Tecnologico della, Sicilia 95121 Catania, Italy; 10 Present Address: Human Technopole, Viale Rita Levi-Montalcini 1, 20157 Milan, Italy

**Keywords:** Cancer models, Cancer stem cells, Experimental models of disease

## Abstract

High Grade Serous Ovarian cancer (HGSOC) is a major unmet need in oncology, due to its precocious dissemination and the lack of meaningful human models for the investigation of disease pathogenesis in a patient-specific manner. To overcome this roadblock, we present a new method to isolate and grow single cells directly from patients’ metastatic ascites, establishing the conditions for propagating them as 3D cultures that we refer to as single cell-derived metastatic ovarian cancer spheroids (sMOCS). By single cell RNA sequencing (scRNAseq) we define the cellular composition of metastatic ascites and trace its propagation in 2D and 3D culture paradigms, finding that sMOCS retain and amplify key subpopulations from the original patients’ samples and recapitulate features of the original metastasis that do not emerge from classical 2D culture, including retention of individual patients’ specificities. By enabling the enrichment of uniquely informative cell subpopulations from HGSOC metastasis and the clonal interrogation of their diversity at the functional and molecular level, this method provides a powerful instrument for precision oncology in ovarian cancer.

## Introduction

High Grade Serous Ovarian cancer (HGSOC) constitutes a major unmet need in oncology as one of the most lethal gynecological cancers, due to the failure of surgery and chemotherapy at eradicating the disease, the ensuing nearly invariable recurrence and very limited therapeutical progress over the past decades [1, 2]. This is, in turn, rooted into the biology of the disease and the technical limitations that have so far hampered its investigation.

The former relate, first of all, to the specific features of the anatomical localization that enable precocious dissemination through the abdomen, with metastatic ascites often concomitant with the first diagnosis. In addition, converging evidence points to the pharmacological resistance of specific subpopulations, varyingly referred to as cancer stem cells (CSCs) or cancer initiating cells (CICs), that can persist after chemotherapy [3, 4] and often remain quiescent for months in the peritoneal cavity from which they fuel renewed and/or continuous growth [5, 6].

Among the technical hurdles that have hampered progress is the inadequacy of available ovarian cancer cell lines to model physiopathologically relevant aspects of the disorder. Not only they do not allow to correlate molecular aberrations to clinical histories [7] and are thus of no use to advance the precision oncology agenda, but they also fail to recapitulate the landscape of alterations observed in most primary tumor isolates [2, 8, 9]. The development of new methods to robustly capture, from the original lesions and in a patient-specific manner, the cell subpopulations that maintain cancer growth is, thus, a key priority in the field.

3D organoid cultures have recently emerged as a powerful modeling approach for a variety of disorders to recapitulate salient features of the original tissue or organ and propagate in vitro relevant subpopulations of cells representative of the original in vivo complexity [10].

Organoids have been derived from several cancer types [11–15], enabling to probe the mutational and functional diversification of individual tumor cells at unprecedented resolution, such as in colorectal cancer [16]. For HGSOC patient-derived organoids have been described only recently, via protocols that aim at capturing and propagating the cellular heterogeneity of the original tumors from which they were derived [17–20]. Such polyclonal organoid platforms do not permit to align the molecular interrogation of individual cancer cells to their effective forming/propagating potential. Here, we thus set out to establish a new method to isolate and grow in 3D the arguably most clinically relevant type of HGSOC cells, namely cells from metastatic ascites, taking advantage of their ability to grow in an anchorage-independent manner [21, 22] and harnessing this property for the establishment of clonal spheroids cultures in individual wells that could thus allow longitudinal tracing of their propagated features. Rather than aiming at the greatest possible recapitulation of the heterogeneity of the original tumor, our approach is intended to rapidly and reliably enrich for the core subpopulation of metastatic HGSOC propagating CICs, making such homogeneously selected cell populations experimentally tractable. Specifically, the single cell-derived metastatic ovarian cancer spheroids (sMOCS) platform here presented is endowed with several advantages over current 3D culture systems used for bulk enrichment of CICs for HGSOC, allowing: (i) the isolation of individual cells from patient’s metastasis to track their propagation potential at a functional and molecular level; (ii) the identification of pathways operating in metastasis in the presence of the optimal microenvironment proxy to in vivo, through ascitic fluid supplementation to the culturing medium.

Importantly, given the specific features of HGSOC, we applied this method directly to metastatic ascites, as a highly informative disease stage of relatively easy access for the streamlined translation of this method to the clinical setting. Finally, we used single cell RNA sequencing (scRNAseq) to define the cellular composition of HGSOC ascites and trace its propagation in both 2D and 3D spheroids culture paradigms, finding that sMOCS recapitulate key features of cancer stemness of the original metastasis that do not emerge from classical 2D culture, including retention of individual patients’ specificities and drug response. Thus, this method establishes the feasibility of enriching physiopathologically relevant cell subpopulation of cancer cells directly from HGSOC ascites and clonally investigating their diversity at the functional and molecular level.

## Results

### Efficient derivation of ovarian cancer spheroids from single cells of metastatic HGSOC ascites

We set out to establish a HGSOC modeling platform to allow: (i) the streamlined and functionally based isolation of CICs; (ii) their growth into 3D monoclonal spheroids; (iii) their serial propagation along with the computational reconstruction of its impact on modeling; (iv) the comparison of 2D and 3D cultures paradigms at single cell resolution to benchmark their ability to recapitulate HGSOC.

A hallmark of HGSOC is the ease with which it precociously metastasizes to the peritoneal cavity, which constitutes a key hindrance to its eradication. This dissemination is accompanied by the production of ascites where cancer cells can be present both as single cells and as floating aggregates. Given the impact of early and diffused metastasis on the poor management of HGSOC, we thus reasoned that ascites would be an accessible and meaningful source of cancer cells for a translationally oriented HGSOC modeling platform.

**Fig. 1:**
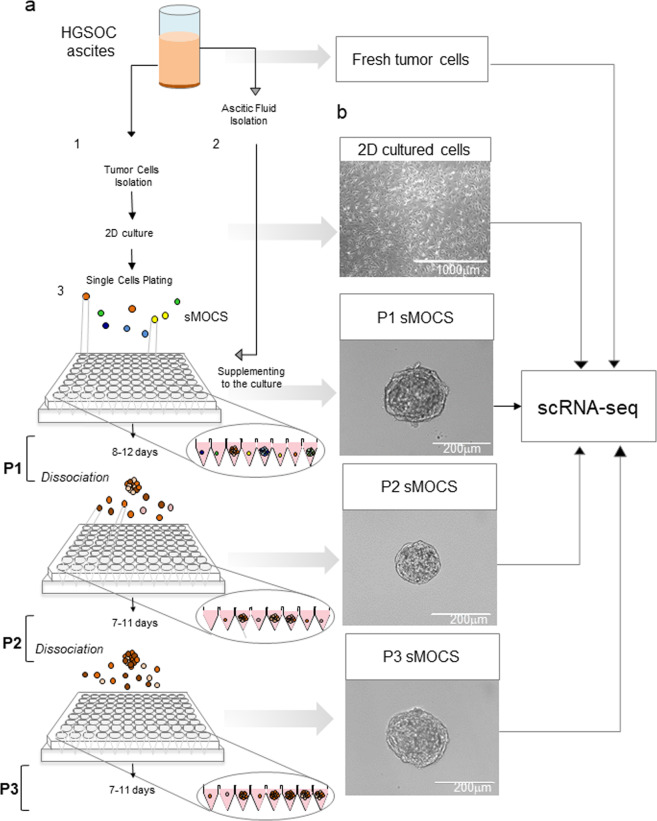
Generation of sMOCS from HGSOC ascites. **a** Scheme illustrating the main steps of the method for generating single cell ovarian cancer spheroids (sMOCS) from HGSOC ascites. Ascitic fluid is centrifuged; the cell pellet is processed for the removal of red blood cells and dissociated as single cell suspension for monolayer culture of tumor cells (step 1); the remaining supernatant after the first centrifugation is processed in order to remove the residual fraction of cells and is used as supplement for growing and culturing sMOCS (step 2). Tumor cells from monolayer culture are dissociated, resuspended in specific culture media for growing single cell from HGSOC ascites and plated by limiting dilution at the density of 1 cell per well in a low-adhesion 96 well V bottom plate (step 3). sMOCS at first passage (P1) are observed after 8–12 days in culture as tridimensional structure of about 200–220 μm of diameter and then propagated through dissociation of a single spheroid in single cells. The single cells are resuspended in the described media and plated as previously indicated in order to obtain the next passages in culture (P2 and P3). The scheme illustrates that fresh ascites, monolayer culture of tumor cells, and sMOCS at different passages are then processed for scRNAseq to obtain their transcriptomic profile. **b** Bright field image analysis (x4magnification for 2D, scale bar 1000 μm; x40 magnification for sMOCS P1, scale bar 400 μm; x20 magnification for sMOCS P2 and P3, scale bar 200 μm).

**Fig. 2:**
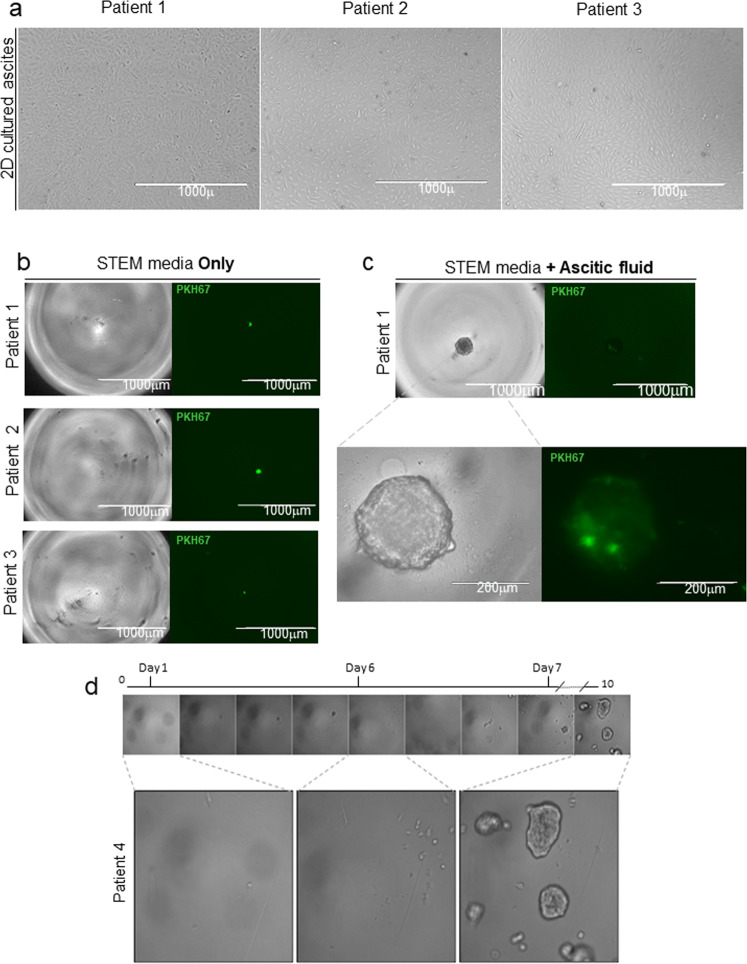
Metastatic ascitic fluid is required for cell proliferation and generation of sMOCS. **a** Bright field images representative of the morphology of primary cultures derived from HGSOC ascites of different patients (x4 magnification for 2D monolayer culture of three different patients, scale bar 1000 μm). **b** Bright field image analysis (x4 magnification for patient 1, patient 2 and patient 3, scale bar 1000 mm) of a single cell from 3 different patients plated in STEM media only and the respective fluorescent signal for PKH67 + (green). **c** Bright field image analysis (x4 magnification for patient 1, scale bar 1000 μm; x20 magnification for patient 1, scale bar 200 μm) of a single cell from patient 1 cultured in STEM media supplemented with ascitic fluid and fluorescent signal for PKH67. **d** Time-lapse of a single well followed during cell division. Day 0: only a single cell is present in the well. Day 2: first mitotic division. Day 4: the small spheroid is formed. Day 5: the small spheroids divide in single cells. Day 6: all the cells derived from the original spheroid start to divide. Day 10: multiple monoclonal spheroids are grown.

To this end, we developed a method for isolating and culturing individual CICs from patients’ metastatic ascites (Fig. 1). CICs, characterized by cancer-maintaining potential, self-renewal and anoikis-resistance, have been isolated from solid tumors and OC mostly by enrichment through tumor-sphere cultures, harnessing their ability to proliferate under non-adherent conditions [21, 22]. For HGSOC, however, no method is yet available for the isolation and culture of CICs. Reasoning that ascites represents a particularly favorable niche for the growth of HGSOC CICs, we supplemented the medium for growing primary cells [23] with cell-free ascitic fluid at different ratios to define its baseline effect (Fig. S1). We observed a significant ascitic fluid-dependent increase in cell proliferation (Fig. S2a, b). Ascitic fluid supplemented to 2D primary culture increases cell proliferation, with the highest efficiency at the 12.5% concentration (Ascitic fluid to medium ratio). We therefore selected this concentration in setting up the optimal condition for the 3D culture of individual HGSOC cells. Specifically, HGSOC ascites from five untreated patients (Table 1) were processed in order to derive 2D cultures of tumor primary cells as described [23] (Fig. 2a). Cells from primary 2D cultures at the first in vitro passage were suspended in ovarian cancer stem cells medium with or without ascitic fluid (12.5%) and plated into 96 wells ultra-low attachment plates at the density of one cell per well. We found that in such non-adherent conditions no sMOCS are generated from single cells plated in medium alone (Fig. 2b), while only the supplementation with cell-free patients’ ascitic fluid enabled the proliferation of individual cells leading to the generation of sMOCS over a course of 8–12 days (Fig. 2c, d). These results indicate that ascitic fluid promotes cell proliferation in bidimensional cultures and is necessary to allow the 3D growth of individually seeded tumor cells.

**Table 1:** Clinical-pathological parameters of patients.

Sample	Diagnosis	Disease state	Grade	Age
Patient 1	Serous surface papillary carcinoma	Primary	3	60
Patient 2	Serous surface papillary carcinoma	Primary	3	46
Patient 3	Serous cystadenocarcinoma	Primary	3	56
Patient 4	Serous surface papillary carcinoma	Primary	3	62
Patient 5	Serous cystadenocarcinoma	Primary	3	58

Overview of the diagnosis, the disease state and the grade of the tumor for each patient with HGSOC from which sMOCS were generated. Age of the patient is referred at the time of diagnosis.

We then aimed to determine the efficiency and the robustness of sMOCS generation across propagation. To ensure the monoclonal derivation of the spheroid, primary cells were stained with the green fluorescent dye PKH67 (cell membrane labeling), in order to be able to trace the single cell and monitor the formation of the spheroid in a single well.

**Fig. 3:**
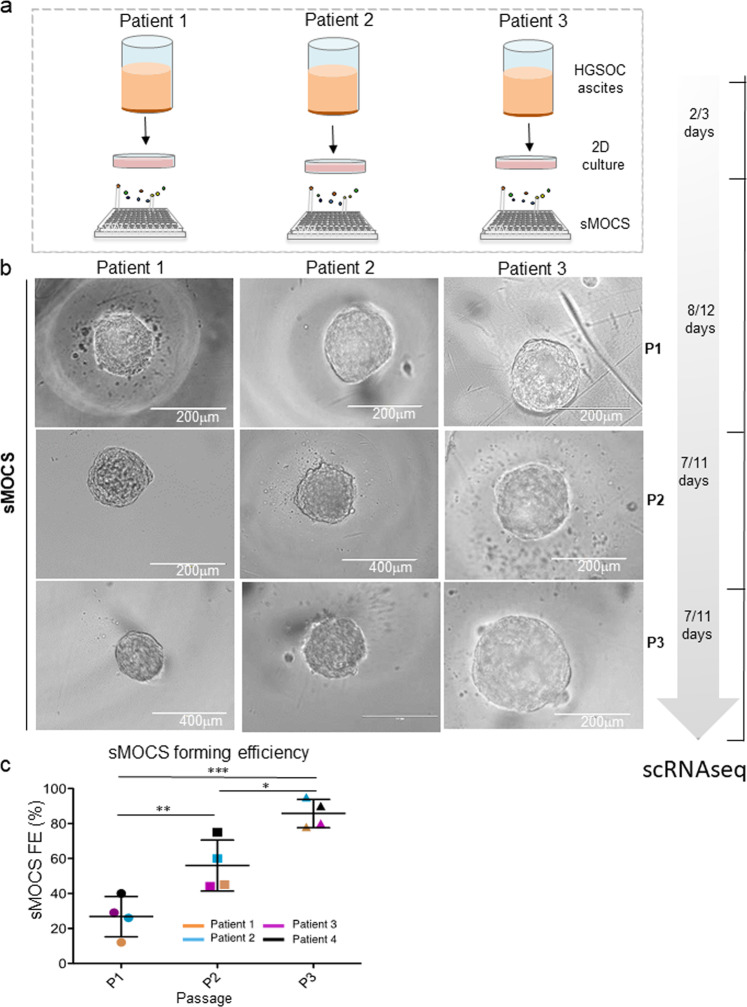
SMOCS forming efficiency increase during passages in vitro. **a** Scheme illustrating the main steps of the culturing method to derive sMOCS with a dedicated timeline indicating the number of days required in culture for each stage. **b** Bright field image of representative images of sMOCS from three different patients at three different passages: P1, P2, and P3, (x20 magnification for sMOCS passage 1(P1) for patient 1, 2, and 3; for sMOCS P2 from patient 2 and 3; for sMOCS P3 from patient 3, scale bar 200 μm); (x40 magnification for sMOCS passage 2 from patient 2 and for sMOCS P3 from patient 1 and 2, scale bar 400 μm). **c** Scatter plot showing the sMOCS forming efficiency (sSFE) as percentage for each patient and for each passage. Primary cultures derived from HGSOC ascites were grown under non-adherent conditions in 96 well plate V bottom in presence of the media supplemented with ascitic fluid to test their ability to generate monoclonal spheroids. The experiment was performed on 4 independent samples. sSFE was calculated as the ratio between number of monoclonal derived spheroids and the number of cells seeded. **d** Graph showing single cell spheroid forming efficiency (sSFE) (mean + SEM) of sMOCS from different patients at passage 1, 2, and 3. P1 *n* = 4, P2 *n* = 4, P3 *n* = 4. Unpaired *t* test, *p < 0.05; **p < 0,01; ***p < 0.001.

To this end, 2D primary cell cultures of patients with HGSOC (Fig. 3a) were labeled with PKH27 and sorted by flow cytometry. The conditions for scaling down the number of cell doublets were optimized to obtain high purity (90–95%) of single labeled CICs (see “Materials and Methods”). Sorted cells were counted and plated by limiting dilution in a 96 well plate and supplemented with 12.5% of patients’ ascitic fluid.

sMOCS at passage 1 (P1) that reached a diameter of about 200–220 microns were dissociated into single cells and plated again in the same conditions for a short-term propagation through passage 2 and passage 3 (P2 and P3, respectively) (Fig. 3b). We observed that each subsequent passage dramatically increased the forming efficiency of sMOCS (Fig. 3c), reaching 90–95% at P3, indicating a progressive enrichment for CICs. Furthermore, by selecting and propagating, across three patients, two different sMOCS at P1 and testing them separately, we also determined different efficiencies of propagation through P2 (Fig. S3), showing that different monoclonal 3D cultures from the same individual and at the same passage capture a gradient of propagation potentials. Finally, we tested the robustness of ascitic fluid supplementation by comparing patient-matched versus patient-unrelated experimental designs and we confirmed their equivalence (Fig. S4).

### Single cell transcriptional dissection of fresh metastatic tumors, 2D and 3D cultures

**Fig. 4:**
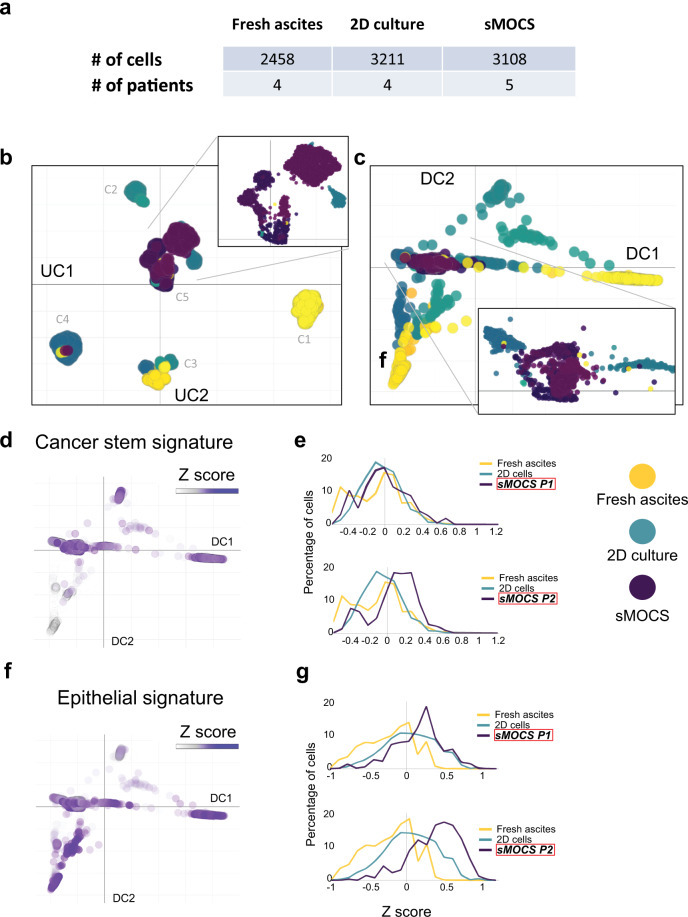
single cell RNAseq reveals enrichment of CICs in sMOCS. **a** Number of cells for samples and patients analyzed after filtering and quality control. **b** UMAP of single cell transcriptomes from cells in (**a**), where each cell is represented by a point; each color tone identifies different conditions among the same patient, yellow tones: fresh ascites; turquoise tones: 2D culture; purple tones: sMOCS;. Top right: the magnification of the central area of the UMAP is enriched mainly for sMOCS cells but also for some fresh ascites and 2D cultured cells. **c** Diffusion map of single cell transcriptomes from cells in (**a**), where each cell is a point; yellow tones: fresh ascites; turquoise tones: 2D culture; purple tones: sMOCS. Bottom right: magnification of the region where all the samples from different patients and conditions converge, enriched in sMOCS cells. **d**–**g** Enrichment analysis of Cancer stem and EMT signature. Left: diffusion maps of all the conditions and patients with a color scale defined by the *z* score of the respective signature; right: frequency plot showing the variation in the distribution of cells as a function of the *z* score of the indicated signature. The higher the percentage of cells with high *z* score, the more enriched the sample is for the indicated signature.

To define the cellular composition of sMOCS at single cell resolution and trace its dynamics over propagation *vis-à-vis* the original metastatic cancer and traditional 2D culture, we performed scRNAseq on a cohort of samples from five patients with HGSOC spanning the following conditions: freshly isolated HGSOC ascites, 2D culture and sMOCS at two different stages of propagation (P1 and P2) (Fig. 4a).

To first obtain a global representation of the dataset, we applied a non-linear dimensionality reduction visualization algorithm, Uniform Manifold Approximation and Projection (UMAP [24]), that has been shown to identify biologically meaningful cell clusters that retain consistency across a broad range of parameters variation, such as metric and number of neighbors. In addition, as an alternative to the t-distributed stochastic neighbor embedding (t-SNE) method, this approach also affords greater preservation of the global data structure [25]. This resulted in the identification of five different cluster of cells (Fig. 4b). Cluster 1 (C1) is composed exclusively of freshly isolated cancer cells, cluster 2 (C2) of primary cancer cells cultured in 2D, cluster 3 (C3) contains both 2D cultured cells and freshly isolated cells, cluster 4 (C4) is mainly composed by 2D cultured cells along with few fresh and sMOCS cells. Last, cluster 5 (C5) comprises cells belonging to all examined conditions, including the majority of sMOCS cells, indicating that they homogeneously select for a specific tumor subpopulation.

To examine the salient properties of our dataset, we then applied diffusion map, a dimensionality reduction method that preserves the underlying structure of the original dataset, thus enabling a meaningful measure of the distances and trajectories intervening across any two given cells [26, 27] (Fig. 4c). Diffusion map reveals a clear tri-partition, with the fresh HGSOC samples (in yellow) widely spread but clearly demarcated from the distributions of the 3D and 2D cultures, (respectively in purple and turquoise, Fig. 4c). This approach also confirmed a continuous relationship across samples represented by the overlap in the distribution of cells among the different conditions, consistent with the fact that freshly isolated cancer cells first undergo a single passage in 2D before being expanded in 3D. Importantly, this unsupervised approach also revealed that the two main components structuring the space of the diffusion map (DC1 and DC2) trace the specificity of the fresh samples from different patients, which however converge towards the region in which sMOCS cells are grouped, underscoring the consistency and homogeneity of features captured by the 3D culture system. Finally, both UMAP and diffusion map allow to draw: (i) a higher variability of 2D cultures compared to sMOCS; (ii) a high degree of consistency across sMOCS passages, pointing to an enrichment in features that are specific and are maintained over time by the 3D model.

### sMOCS capture both general and patient-specific molecular features of HGSOC ascites

**Fig. 5:**
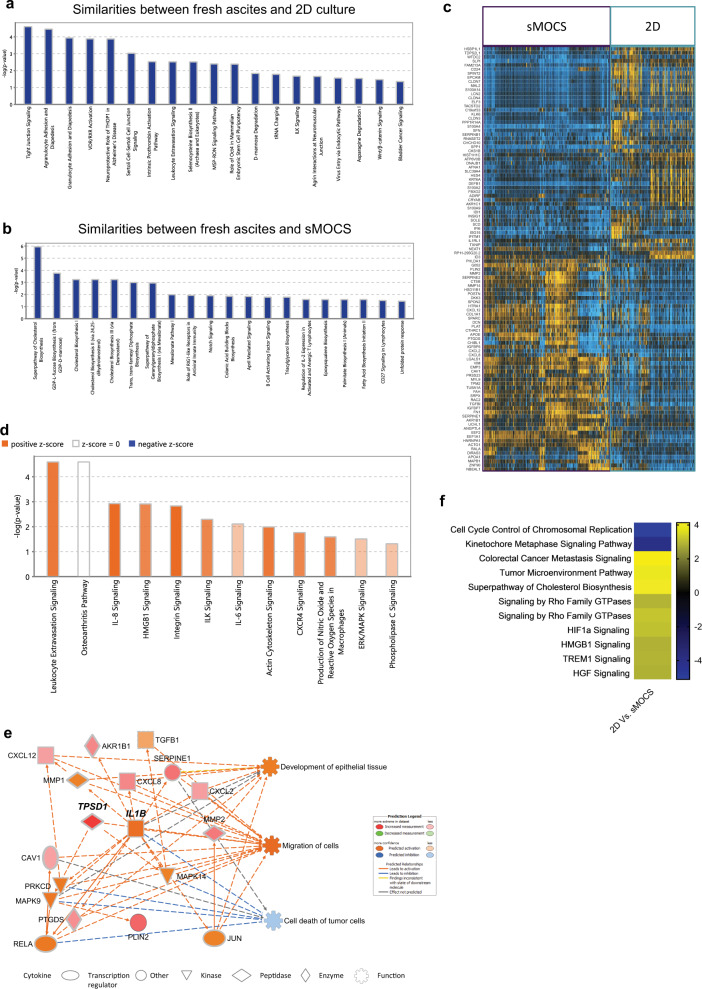
sMOCS capture relevant features of the fresh tissue while preserving hallmarks of HGSOC ascites that do not emerge from 2D culture. **a** Pathway analysis on the groups of genes and 2D culture; (**b**) pathway analysis on the groups of genes that correlate among fresh ascites and monoclonal 3D system; (**c**) heatmap of differentially expressed genes between sMOCS and 2D culture; (**d**) pathway analysis performed on DEGs in (**c**): color scale define the predicted activation/deactivation of the pathway; orange identifies the pathways activated in sMOCS; (**e**) IPA causal network derived from DEGs in (**c**): color scale define the expression of downstream regulated genes and their associated function, the orange tone identifies the genes and functions activated in sMOCS.

In order to investigate the cellular composition of sMOCS, we took advantage of validated cell type-specific gene markers to define transcriptional signatures to interrogate our dataset. In particular, to assess whether our system is enriching for CICs, we employed markers that have been widely used for their isolation in solid tumors and specifically in HGSOC [22] (Table S1). Moreover, considering that more than 90% of malignant ovarian tumors have an epithelial origin and that epithelial mesenchymal transition (EMT) is both a crucial factor for cancer progression and a prerequisite for metastatization [28, 29], we also investigated the expression of EMT-associated genes along with those defining the epithelial compartment per se [30, 31] (Table S1). The transcriptomic comparison between fresh metastatic samples, 2D and 3D cultures at different passages revealed that sMOCS retain a consistent subpopulation of CICs (Fig. 4d), as measured by the *Z* score of the cancer stem signature (Table S1). Interestingly, the number of cells with higher levels of expression of this signature is notably increased in P2 when compared to fresh tumor cells, 2D and sMOCS at P1, underscoring the progressive enrichment for stem cell features through propagation of the 3D model (Fig. 4e). Furthermore, while we observed a higher expression of epithelial cells’ markers in fresh cancer cells when compared to 2D and sMOCS (Fig. S5a, b), we found that the expression of EMT-related markers already increased in P1 sMOCS, as compared to fresh cells and 2D cells, further augmented in P2 (Fig. 4f–g). To exclude that sMOCS enrichment in CSCs was due to the supplementation of ascitic fluid to the 3D culture rather than to the culturing method per se, we compared at the transcriptional level (i) sMOCS (cultured with ascitic fluid as previously shown), (ii) 2D cells cultured in the standard medium (2D-M) and (iii) 2D cells cultured in medium + ascitic fluid (2D-AS) obtained from 4 HGSOC patients’ ascites. Bulk RNAseq analysis showed that 39% of the transcriptional variance in this dataset was strictly dependent on the two culturing methods used, i.e., 2D vs sMOCS, rather than on the supplementation of ascitic fluid per se (19% of variance) (Fig. S6a). Moreover, by differential expression analysis, we could score a differential impact of ascitic fluid on gene expression of 2D cells and spheroids (Fig. S6b). Indeed, 2D cells showed an upregulation of genes controlling cell cycle-related pathways, while sMOCS showed an activation of genes involved in stem-related pathways, independently of the baseline condition used for the comparison, i.e., 2D cells cultured with/out ascitic fluid (Fig. 5f and Fig. S6d). This is also supported by the almost complete overlap between differentially expressed genes when comparing 2D-AS or 2D-M Vs sMOCS (Fig. S6c). These findings demonstrate that the activation of stem-related pathways is not a generic byproduct of ascitic fluid supplementation but a specific feature revealed by the sMOCS culture system.

We thus conclude that the sMOCS culture system, in contrast to 2D cultured cells, retains and progressively enriches for cell functional features of the fresh ascites related to cancer stemness and to the EMT process.

Finally, we sought to determine whether sMOCS could retain also patient-specific features, despite the overall convergence observed through diffusion map (Fig. 4c). To this end, we performed a differential expression analysis in the whole dataset to identify genes that are differentially expressed in at least one of the patients irrespective of experimental condition [32]. We identified 659 differentially expressed genes (DEGs), a subset of which, comprising 97 genes (15%), was retained between fresh ascites and sMOCS (Fig. S7a). This suggests that sMOCS, despite being less variable than fresh and 2D culture, retain a consistent portion of the gene expression profile characterizing each individual patient’s tumor. To confirm this result with an unsupervised approach, the same dataset was analyzed by weighted gene co-expression network analysis (WGCNA), selecting the gene modules most strongly associated to individual patients, regardless of condition (Fig. S7b). This led to the identification of 3 modules (Fig. S7b, blue, green, and pink) which were then visualized by UMAP (Fig. S7c). As shown in Fig. S7c, d, UMAP clearly identifies patient-defining clusters that, importantly, comprise cells across all three conditions (fresh ascites, 2D and 3D), thereby confirming that sMOCS are able to retain and propagate a salient portion of patient-specific cancer-associated gene expression.

### sMOCS highlight features of HGSOC ascites that do not emerge from 2D culture

To assess whether sMOCS constitute a relevant and possibly superior alternative to classical 2D culture, we verified which features of the original fresh acites were maintained throughout spheroids’ propagation *vis-à-vis* the 2D counterpart. To test this, we used WGCNA to identify gene modules that correlate between conditions (Fig. S8), focusing on the features that are maintained between fresh ascites and 2D culture, and between fresh ascites and sMOCS. Among the identified modules none showed a strong correlation between 2D and sMOCS, whereas several were correlated between the fresh ascites and, respectively, either the 2D or the 3D culture paradigms. To probe such similarities at the level of biological pathways, we applied gene ontology enrichment analysis on gene modules of either correlation. The analysis on the gene modules whose transcriptional behavior is shared between fresh ascites and 2D cultures revealed an enrichment for pathways related to signal transduction that regulate cell proliferation and gene expression [33], diapedesis [34], activation of vitamin D receptor pathway (VDR/RXR) [35, 36], as well as activity of integrin-linked kinase ILK [37]. These pathways have been involved in regulation of tumor growth, including in OC. In addition, we found enrichment for the octamer binding transcription factor 4 (Oct-4) pathway, which regulates stem cell self-renewal and pluripotency with emerging roles in regulating tumor initiating cells [38, 39], and for the Wnt beta-catenin signaling pathway (Wnt/β), involved in stem cell regeneration and organogenesis [40] (Fig. 5a). In contrast, an ontology analysis of the genes defining the similarity between fresh ascites and sMOCS showed an enrichment in pathways related to the biosynthesis of cholesterol and triacyl glycerol biosynthesis. This is consistent with previous observations showing that abnormal expression levels and mutations of genes involved in the cholesterol homeostasis and lipid metabolism are related to cancer [41, 42] and OC [43, 44]. Likewise, Notch signaling, that regulates cell proliferation, stem cell maintenance and that plays a critical role in the cross talk between angiogenesis and CICs self renewal, was also an enriched pathway defining the similarity between fresh ascites and sMOCS cells [45] (Fig. 5b). Thus, our WGCNA across conditions indicates that while 2D cultures recapitulate well-known biological pathways implicated in cell proliferation and tumor growth, sMOCS highlight additional features especially related to the metabolic and signaling state of the fresh metastatic samples that had so far resisted in vitro tractability.

To identify the specific differences between sMOCS and 2D cultured cells, we performed differential expression analysis between these two categories, identifying 104 DEGs common across all patients (Fig. 5c). Functional analysis of this set of genes by Ingenuity Pathway Analysis (IPA) [46] revealed an upregulation of pathways related to interleukin 8 (IL8) [47] and integrin signaling in sMOCS, both correlating with tumor growth and progression (Fig. 5d). Next, to investigate the upstream biological causes and predicted downstream effects of such differentially regulated circuits, we applied IPA causal network analysis (Fig. 5e) and uncovered in sMOCS the following two key insights: (i) a downregulation of processes related to cancer cell death mediated through the action of v-rel avian reticuloendotheliosis viral oncogene homolog A (RELA/P65), mitogen activated protein kinase 9 and 14 (MAPK9, MAPK14), protein kinase C delta type (PRKCD) and interleukin 1 beta (IL1B); (ii) an up-regulation of the functions related to the development of epithelial tissue and cell migration, mainly mediated by IL1B, C-X-C motif chemokine 8 and 12 (CXCL8, CXCL12) and tumor growth factor beta 1 (TGFB1). The same results have been confirmed from bulk RNAseq performed on 2D primary cells and sMOCS: DEGs between sMOCS and 2D cells principally relied on genes involved in cholesterol biosynthesis and pathways related to tumor progression (i.e., Hypoxia Inducible Factors (HIFs) signaling genes have been shown to stimulate Notch and Oct4 pathways which control stem cell self-renewal and multipotency [48]; high mobility group box-B protein (HMGB) that promotes tumor growth and metastasis in epithelial OC [49], IL-6/JAK/STAT3 pathway which is involved in proliferation, invasiveness and metastasis of tumour cells [50]) confirming the ability of sMOCS in enriching for CICs (Fig. 5f).

### sMOCS reproduce the heterogeneity of response of individual tumor cells to drug treatment

**Fig. 6:**
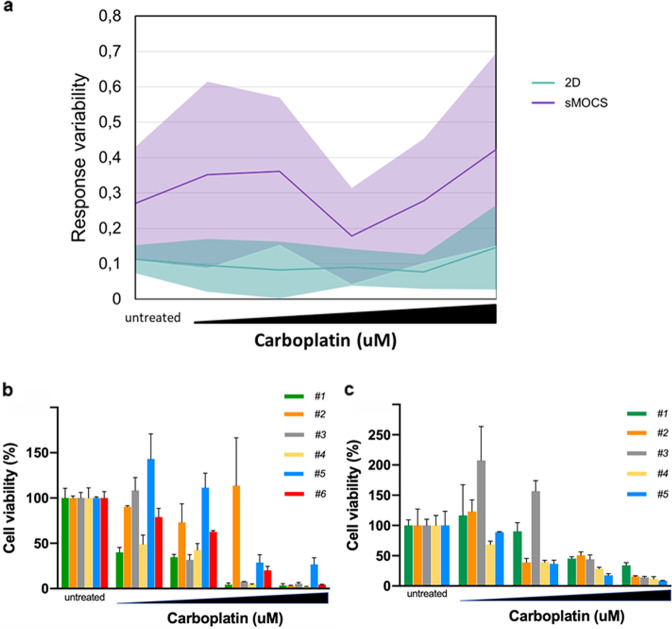
Drug treatment of sMOCS highlights inter- and intra-patient variability in response. **a**, **b** Comparison of the response of sMOCS vs 2D cultures to drug treatment. sMOCS and 2D cultures were treated with carboplatin (untreated, 5 µM, 10 µM, 25 µM, 50 µM, 75 µM). Cell viability was measured with CellTiter-Glo^®^ 3D Cell Viability Assay (for sMOCS) and CellTiter-Glo^®^ Luminescent Cell Viability Assay (for 2D cultures). **a** Results obtained from three independent naive patient samples, shown as means + SD. *P* value was calculated by a two-tailed unpaired Student’s *t* test, and the difference among monoclonal-derived spheroids and 2D cultures resulted statistically significant: 0.0262. **b** Data obtained from cells derived from a patient sample after chemotherapy (post-chemotherapy), at relapse, shown as means + SD. *P* value was calculated as above: 0.0454. **c**, **d** Intrapatient heterogeneity of response to drug treatment using sMOCS. P2 sMOCS were dissociated and cells seeded at 500 cells/well, and then derived spheroids were treated with carboplatin (untreated, 50 µM, 100 µM, 150 µM, 200 µM) and measured for cell viability as described above. In (**c**), spheroids at P3 derived from six cancer initiating cells of patient 17AS27 were analysed, in (**d**) spheroids at P3 derived from five cancer initiating cells of patient 18AS18. *P* values were determined by a two-tailed unpaired Student’s *t* test. In (**c**), the following pairs showed a significant *P* value: #1 vs #2: 0.0152, #2 vs #4: 0.0304. Comparison of the response of sMOCS vs 2D cultures and intrapatient heterogeneity of response to drug treatment. **a** After 7 days, cell viability was measured.Response variability shown as percentage error between sMOCS/2D cells derived from 6 patients. **b**, **c** Spheroids were treated with carboplatin (untreated, 50 µM, 100 µM, 150 µM, 200 µM) and measured for cell viability. In (**b**), spheroids derived from six individual cell clones of patient 17As27 were analysed, in (**c**) spheroids derived from five individual cell clones of patient 18As18.

Most OC patients initially respond to therapy with platinum compounds, but eventually tumors progress and acquire drug resistance. We compared the sensitivity to carboplatin of sMOCS versus 2D cultures derived from 6 patients diagnosed with HGSOC and not previously subjected to any drug treatment. Interestingly, while 2D cells presented only slight variations in cells’ viability among samples from different patients, showing in almost all of them a dose-dependent decrease, we recorded highly different responses to carboplatin on sMOCS derived from different patients (Fig. 6a). This evidence suggests that sMOCS, already able to capture patient-specific features at the single-cell transcriptomic readout, are sensitive enough to preserve them also at the level of drug response, highlighting their potential as patient-matched system for a personalized treatment. Moreover, different scores in cells viability were recorded also among sMOCS derived from the same patient at the same drug concentration (Fig. 6a), suggesting that sMOCS, differently from the 2D cultures, are also able, being monoclonal, to capture intra-patient functional differences across individual ascites cells.

Finally, to confirm that sMOCS provide an experimental model to tackle intra-tumor heterogeneity, we derived P2 sMOCS from two additional patients, and we seeded spheroids (thus originating from single tumor cells) for drug treatment studies. For both patients, treatment with increasing doses of carboplatin revealed that different spheroids from the same patient exhibited differential sensitivity to treatment, with some spheroids responding even at the lowest doses while others proving resistant to treatment even at the highest dose (Fig. 6b, c). These results highlight the value of sMOCS in revealing the cellular heterogeneity of metastatic ascites through scalable and functionally relevant assays.

## Discussion

Here we describe a short-term culture system that captures the in vitro potential of CICs from HGSOC metastatic ascites and amplifies it to make it experimentally tractable.

The method was designed to capture, from the diversity of cell subpopulations that characterize metastatic samples, the range of individual cancer cells that are able to propagate cancer growth in 3D, thereby enabling the functional and molecular interrogation of metastatic ascites’ heterogeneity.

Our method presents the following key innovations.

First, we found that, across patients, ascitic fluid supplementation is strictly required to enable the growth of individual tumor cells into sMOCS and their propagation, pointing to the functional relevance of components shared among metastatic ascites samples. Thus, while the identification of the specific molecule(s) mediating this effect will be an active area of mechanistic investigation, the method can be immediately implemented harnessing the generalized effect of ascitic fluid batches in non patient-matched settings.

Second, the molecular characterization of sMOCS compared to fresh HGSOC ascites and 2D cultures through scRNAseq revealed that sMOCS retain and amplify, differently from 2D cultures, specific cell subpopulations from metastatic samples and that these reveal distinct and thus far undetected cancer stem-related functional specificities (Fig. 5). Importantly, sMOCS also retain a significant portion of the inter-patient molecular diversity detected in the fresh ascites (Fig. S7), highlighting them relevant as sensitive patient-matched avatars to advance precision oncology in the HGSOC field, both in terms of prognostic markers and druggable vulnerabilities.

Third, the design of the sMOCS derivation set up provides clear edges over current approaches that have been interrogating OC stemness relying on the generation of spheroids cultured in bulk [22]. A specific hallmark of OC is indeed the intraperitoneal metastatic route that cancer cells tread through the intra-abdominal fluid-filled space where they can survive either as single cells or as multi-cellular aggregates (MCAs) [51]. That such individual cells can persist and fuel metastatic growth and/or relapse underscores the need to uncover their properties at clonal resolution. In this respect, the scalability of our method affords particular advantages, being uniquely suitable for single-cell high throughput plating, using microwells or micropillars [52, 53], thus optimizing the sample yield per experiment and streamlining patient-specific drug screenings. In turn, this allows the multi-omic characterization, at scale, of the differential response to treatment of CICs, increasing statistical power and resolution for both inter- and intra-patient study designs. Indeed, the observation that sMOCS capture both inter- and intra-patient tumor heterogeneity lays down the foundations, in HGSOC, for probing at the highest resolution patient-specific tumor permissive features, including drug resistance, through robustly propagated in vitro avatars. Together, our results demonstrate the power of sMOCS in furthering the mechanistic dissection of metastatic HGSOC by aligning clinical epistemology to physiopathologically meaningful, experimentally tractable patients’ models.

## Materials and methods

### Samples

Ascites samples were obtained upon informed consent from patients undergoing for surgery treatment for primary, not recurrent HGSOC, at the Gynecology Division of the European Institute of Oncology, IEO, Milan, Italy. Table 1 contains the list of samples related to the patients diagnosis.

### Ethics approval

The study was conducted upon approval of the Ethics Committee of the European Institute of Oncology, IEO, Milan, following its standard operating procedure (“presa d’atto” from 24/7/2017). Only tissue samples from patients who have given informed consent to (i) the collection of samples for research purposes and their storage into the Biobank of the European Institute of Oncology and (ii) the transfer of samples to other research institutions for cancer research purposes have been used in this project. Collected personal data have been pseudonymized, and have been stored and processed in compliance with the applicable data protection legislation, D.Lgs 196/2003 and, since 25 May 2018, Regulation (EU) 2016/679 (General Data Protection Regulation).

### Primary tumor cell culture

We isolated epithelial ovarian cancer (EOC) cells from metastatic peritoneal ascites of 9 patients according an already established protocol [23]. To derive primary tumor cells, obtained ascites were transferred in to polypropylene 750 ml Bio-bottle (Thermo-scientific cat.no. 75003699) centrifuged at 300 x g for 5 min, the supernatant was harvested for downstream processing, while the cells pellet was resuspended with ACK lysing buffer (Lonza, cat.no. 10–548E) and incubated for 5 m at RT to lysate red cells. Tumor-derived cells were cultured on collagen-I-coated cell culture flasks 75 cm^2^ (Corning BioCoat, cat.no. 354485) in CO^2^ incubator at 37 °C.

### Ascites processing

According to the volume, the supernatant of the ascites obtained following the first centrifugation for the derivation of primary cells is transferred in 50 ml polypropylene tubes and centrifuged at (1900 x g) 3000 rpm for 30 m at RT in order to remove all residual cells [54]. The supernatant is collected and transferred into a disposable sterile filter system with bottle of 500 ml and or 1000 ml volume with a PES membrane of pore size of 0.22 micron for vacuum filtration (Sartorius stedim, Sartolab BT filter system cat.no.180C2, 180C3) while the residual pellet is discarded. The filtrate is directly used for experiments, or collected as working aliquotes in 15 ml tubes and stored at −80 °C. After thawing, the filtrate is moved into a 50 ml tubes and filtrated again with a vacuum driven sterile filter of 50 ml volume (Millipore, Steriflip cat. no. SCGP00525) through a suction canister soft liners (Medline, MED-SOFT disposable pre-gelified liner).

### Generation of single cell metastatic ovarian cancer spheroids from HGSOC ascites

Primary tumor cells at passage 1 were dissociated through trypsin/EDTA (Lonza cat. no. BE17–161F), centrifuged at 300 x g for 5 min, the supernatant was removed and the cell pellet was washed twice with pre-warmed Dulbecco-s Phosphate Buffered Saline (D-PBS 1X) and incubated with 0.25% trypsin/EDTA solution at 37 °C until cells are detached from the bottom of the flask. Cells were collected in D-PBS, pelleted at 300 x g for 5 m at RT, suspended in 500 μl of pre-warmed stem media (MEBM) and counted both with TC-20 automated cell counter (Bio-Rad) and counting chamber (Biosigma-Fast Read 102).

The cells were centrifuged at 300 x g for 5 m, and the pellet was resuspended in pre warmed serum-free MEBM supplemented with 100 U/ml penicillin, 100 ug/ml streptomycin, 2 mML-glutamine, 5 ug/ml insulin, 0.5 ug/ml hydrocortisone, 1 U/ml heparin, 2% B27, 20 ug/ml epidermal growth factor, 20 ug/ml fibroblast growth factor and diluted by serial limiting dilution at the density of 1 cell for well in the media supplemented with 12.5% of ascitic fluid for plating into low cell adhesion 96 well plates (Sumitomo Bakelite cat. no. MS-9096V 96 well) with a final volume of 200 μl for well.

Cells were monitored daily by microscope visualization, until spheroids formation at passage 1. The time required for the growth of sMOCS is sample dependent from 7 to 12 days. No changing media is required, however if liquid evaporation occurred we supplemented 25–50 μl of fresh media plus ascetic fluid. Percentage of single cell derived spheroid forming efficiency (SFE) was calculated for every passage as the ratio between the total number of spheroids generated in a 96 well plate, and the number of cells seeded and expressed as percentage.

### Propagation of sMOCS in vitro

For propagation at P2 or P3, a single sMOCS at day 8–11 was moved from 96-well V-bottom ultra-low attachment plates to 48-well ultra-low attachment plates (Corning), and incubated with 400 μl of pre-warmed 0.25% trypsin/EDTA solution at 37 °C for 20–30 m with gently pipetting for 20 times every 8 m and visualized by microscope for the stadium of disaggregation. To avoid loose of cells the tip was always rinsed with medium before pipetting. Cells were harvested, washed once in MEBM plus ascitic fluid and centrifuged at 300 x g for 5 min. Supernatant was discarded and the pellet of cells was re-seeded in 96 well plate as previously described in order to obtain pasage 2, P2, which requires a culturing time of about 7–9 days. The same procedure was applied to generate sMOCS at passage 3, a tridimensional structure formation was observed also between 7–9 days. Percentage of spheroidsspheroid forming efficiency was calculated accordingly.

### PKH67 staining

Epithelial adherent cells were dissociated as single cells, harvested, washed with MEBM media (Lonza cat. no. CC-3151) without serum centrifuged at 300 x g for 5 min. Cells were counted through with TC-20 automated cell counter (Bio-Rad). Cells were resuspended in a final working volume of 2 ml following the procedures for general cell membrane labeling for PKH67 (Sigma–Aldrich cat. no. PKH67GL). We scaled-down the number of cells necessary for the staining in the final working volume of 2 ml to 100.000 cells; EDTA 0.01% was added to the working. Cells were suspended in 2 ml of MEBM plus 0.01% EDTA.

### Imaging

Image. Images were taken with EVOS Cell Imaging Systems, and images taken in Fig. 2d were acquired in Leica SP5 confocal microscope with 10x objective. Images were taken every day for the first 9 days directly from the 96 wells. During the acquisition’s interval the samples were store in the incubator. Images were reconstructed by a custom script using python and OpenCV.

### FACS analysis

The percentage of positive stained PKH67 cells was measured by BD Influx Sorter (BD Biosciences). Positive stained cells were recovered after sorting in falcon round bottom polystyrene tubes (STEMCELL technologies cat.no. 38007) containing 5 ml MEBM plus 0.1% FBS.

### Single cell preparation, cDNAsynthesis generation of single cell GEM and libraries construction

Fresh cells from ascites, 2D primary cells and single cell derived spheroids were dissociated in order to obtain single cells suspension. SMOCS were collected at day 10, and 20–25 spheroids for passage and for patient were dissociated by incubation with a solution 0.25% of trypsin/EDTA at 37 °C for 20 m. Cells in suspension were washed with D-PBS 1X and centrifuged at 300 x g for 5 min. The cell suspension was passed once through cell strainer (Bel-Art; Flowmi cell strainer for 1000 microliter pipette tips, cat. no. H13680–0040) to remove cellular debris and clumps and was resuspended with wide bored tip in D-PBS 1X supplemented with 0.04% bovine serum albumin BSA, Sigma. The cell concentration was determined through TC-20 automated cell counter (Bio-Rad). Droplet-based single cell partitioning to generate single cell gel beads in emulsion (GEMs) was obtained by loading the appropriate cell dilution onto 10x Genomics Single Cell 3’Chips mixed with reverse transcription mix using the Chromium Single-cell 3’ reagent kit protocol V2 (10x Genomics; Pleasanton, CA), according the manufacturer’s protocol. The gel beads are coated with unique primers bearing 10× cell barcodes, unique molecular identifiers (UMI) and poly(dT) sequences.The single-cell suspension at a density of 1000 cells/μl was mixed with RT-PCR master mix and loaded with Single-Cell 3′ gel beads and partitioning oil into a single-cell 3′ Chip. The chip was then loaded onto a Chromium instrument for single-cell GEM generation within which RNA transcripts from single cells are reverse-transcribed. Barcoded full-length cDNA are generated using Clontech SMART technology. cDNA molecules from one sample were pooled and preamplified. The amplified cDNAs were fragmented. Final libraries were incorporated with adapters and sample indices compatible with Illumina sequencing, and quantified by real-time quantitative PCR (calibration with an in-house control sequencing library). The size profiles of the sequencing libraries were examined by Agilent Bioanalyzer 2100 using a High Sensitivity DNA chip (Agilent). Two indexed libraries were equimolarly pooled and sequenced on Illumina NOVAseq 6000 platform using the v2 Kit (Illumina, San Diego, CA) with a customized paired end, dual indexing (26/8/0/98-bp) format according the manufacturer’s protocol. Using proper cluster density, a coverage around 250 M reads per sample (2000–5000 cells) were obtained corresponding to at least 50.000 reads per cell.

### Bulk RNA sequencing

2D primary cells from 4 samples’ patients have been cultured in medium. Then, cells have been divided into 2 conditions (i) 2D cells cultured in standard medium and (ii) 2D cells cultured in medium + 12.5% of ascitic fluid obtained from the same patient. After 3 passages, 2D cells have been used to perform total RNA extraction through RNeasy Mini Kit, QIAGEN. From the same samples, we obtained sMOCS at passage 2. After the dissociation of spheroids in single cell as described in Propagation of sMOCS in vitro, total RNA extraction has been performed, using the same kit. 200 ng of RNA were subjected to library preparation with the Illumina Truseq stranded total RNA kit. Sequencing has been performed to obtain an average of 20 million reads per sample. Raw data were aligned to genes through the salmon pipeline. Differential expression analysis was performed with the edgeR package, and differentially expressed genes were selected using logFC > 1 and FDR < 0.01 as thresholds. Ingenuity Pathway Analysis (IPA) was used for enrichment and regulator effect analyses.

### Test of sensitivity to carboplatin of sMOCS versus 2D cultures

Adherent cells were seeded at the density of 500 cells/well in 96 well white flat bottom assay plates (Costar) and, after 24 h from the seeding, were treated for 7 days with carboplatin. sMOCS were seeded one for each well in ultra-low attachment 96 well black with clear round U-bottom spheroid microplates (Corning) and only those having same size were included in the assay. The treatment was performed at the same manner of adherent cells and viability was measured through CellTiter-Glo^®^ Luminescent Cell Viability Assay (Promega). All the results obtained from 2D cells and sMOCS have been normalized on the corresponding untreated condition, and mean and standard error have been calculated for each patient. Then, error percentage has been calculated considering mean and standard error for both 2D primary cells and sMOCS.

### Test of sensitivity to carboplatin of sMOCS from two patients

P2 sMOCS were dissociated and the cells seeded at 500 cells/well in ultra-low attachment 96 well black with clear round U-bottom spheroid microplates (Corning). 72 h after the seeding, spheroids were treated with carboplatin. After 72 h from the beginning of treatment, viability was measured through CellTiter-Glo^®^ 3D Cell Viability Assay (Promega).

### Statistical analysis

Statistical analyses were performed using PRISM (GraphPad, version 6.0). Statistical significance was tested with the unpaired (nonparametric) *t* test. *N*, *p* values, and significance are reported in each figure and legend. All results were expressed as means ± SD.

### Data analysis

Single cell sequenced libraries were aligned with CellRanger pipeline, we used Hg38 for indexing reference transcripts. Resulting data was imported in python as anndata object. We used scanpy vs 1.3.1 and pandas for downstream analyses in python. Normalization was performed following Seurat [55] pipeline.

For graphs in Fig. 4d we calculated *z* score considering all the cells regardless of the condition. In the left panels we kept the diffusion coordinates and considered the same color scale for all the graph. The same *z* score was used in the frequency plot on the right, plotted data was normalized dividing by the area under the curve.

Single cells data was than clustered by diffusion distance to obtain more coverage and more consistent results for the downstream analyses. Final number of cells per cluster is between 20 and 40, these numbers were chosen to minimize the differences in number of reads per cluster and in order not to lose the heterogeneity of the system. Data was imported in R and Gene groups were identified with WGCNA (with minClusterSize 42 and SoftPower 5) considering among the Module-Trait Relationships (MTRs) those with high correlation between patients or conditions.

Differential expression was performed with edgeR, filtering genes by FDR < 0.05 and logFC > 1.25, to keep all the possible information for the downstream analysis (gene ontologies, pathways analysis, etc).

We used a combination of Causal Network Analysis, Downstream Effects Analysis, Upstream Regulator Analysis and Molecule Activity Predictor from Ingenuity Pathway Analysis [46] to identify the impact of the genes identified from differential expression or from correlation network analysis.

Heatmaps were generated from logCPM using a modified version of Clustergrammer [56], hierarchical clusterings included were performed considering correlation as distance.

## Supplemental experimental procedures

### Calculation of single spheroid forming efficiency (SFE)

Percentage of single cell derived spheroid forming efficiency (SFE) was calculated for every passage as the ratio between the total number of monoclonal spheroids generated in a 96 well plate, and the number of cells seeded and expressed as percentage.

### MTT assay

3-(4,5-dimethylthiazolyl-2)−2,5-diphenyltetrazolium bromide or MTT (M 5655 Sigma–Aldrich) assay was used for measuring cell metabolic activity and to assess cell proliferation rate of primary tumor cells. Primary cells were plated and grown in medium for primary epithelial tumor cells at different density on 96 well plate. After 48 h cells were incubated for 3 h with MTT reagent at 37 ^o^C and then treated with MTT solvent at RT for 5–10 m. Absorbance was measured at OD = 590 nm.

## Supplementary information

Figure S1

Figure S2

Figure S3

Figure S4

Figure S5

Figure S6

Figure S7

Figure S8

Table S1

## Data Availability

Bulk and single cell RNAseq data generated and analyzed in the paper are available in the ArrayExpress repository, under the accession numbers E-MTAB-11194 and E-MTAB-11207, respectively.

## Code availability

All code used for analysis is available from the corresponding author upon request.
