# Supplementary figures and images for: Single cell-derived spheroids capture the self-renewing subpopulations of metastatic ovarian cancer
Source: Cell Death Differ. 2021 Nov 29;29(3):614–26. doi: 10.1038/s41418-021-00878-w (PMC8901794; doi:10.1038/s41418-021-00878-w)

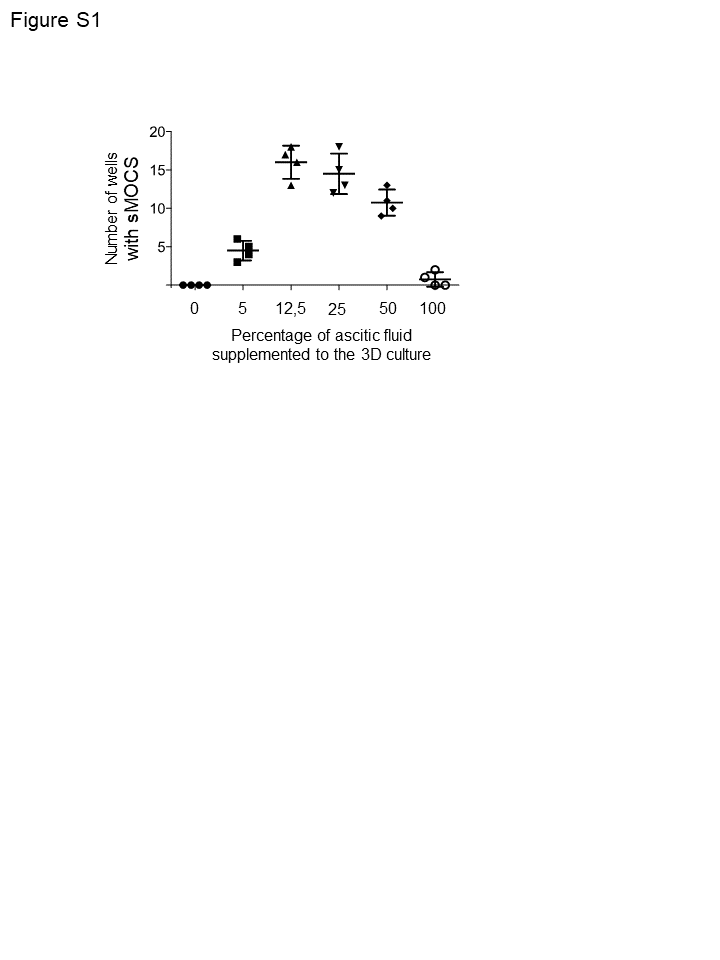

Supplement: Supplementary file 1 — Figure S1 [file 41418_2021_878_MOESM1_ESM.png]

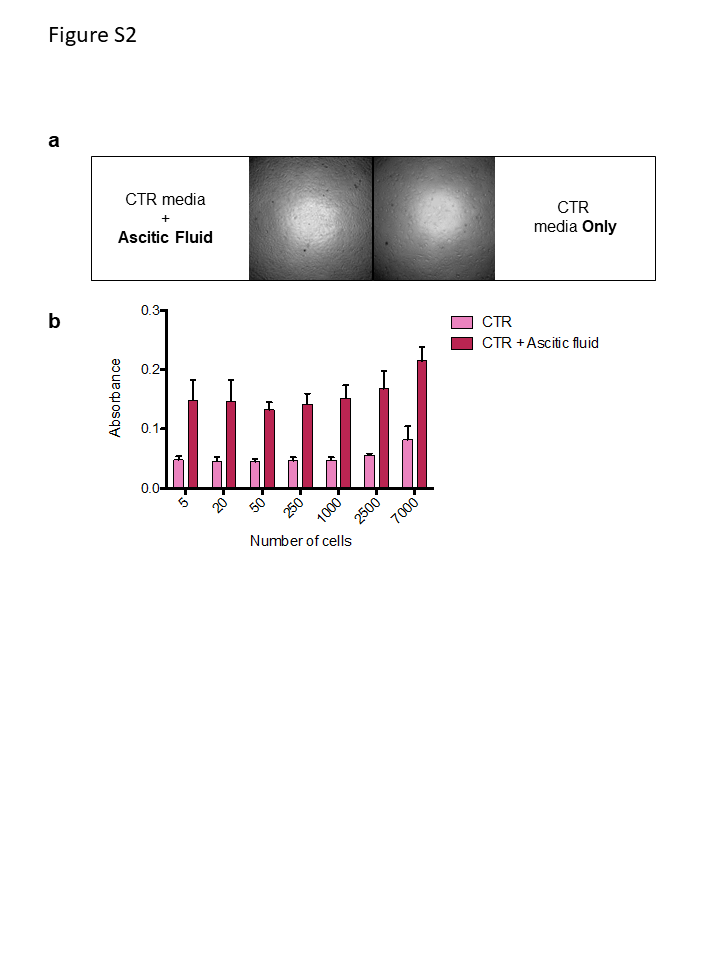

Supplement: Supplementary file 2 — Figure S2 [file 41418_2021_878_MOESM2_ESM.png]

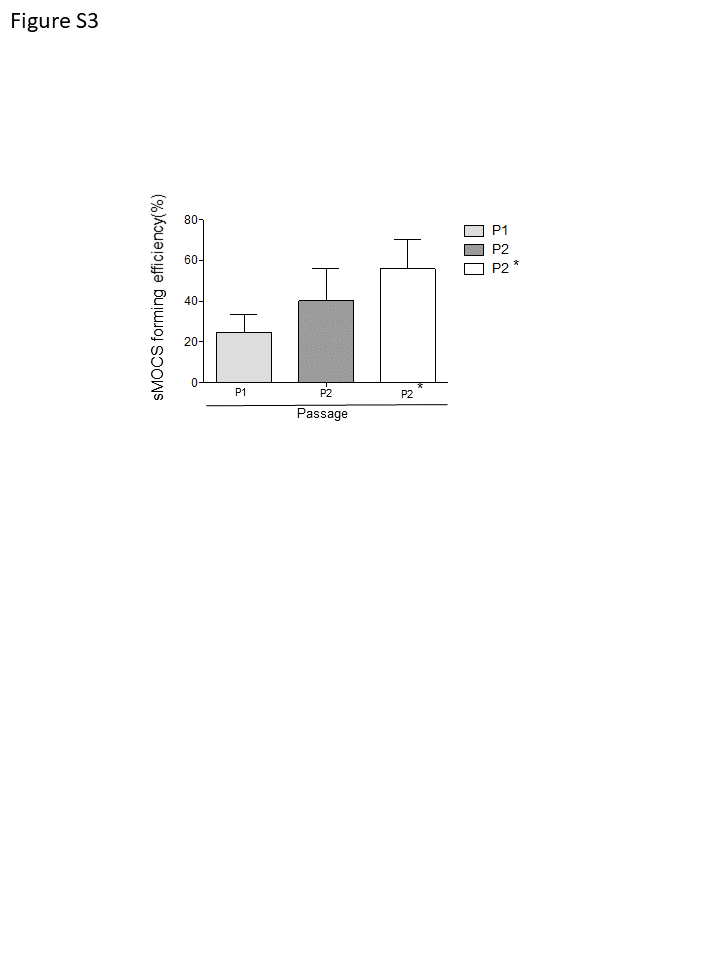

Supplement: Supplementary file 3 — Figure S3 [file 41418_2021_878_MOESM3_ESM.png]

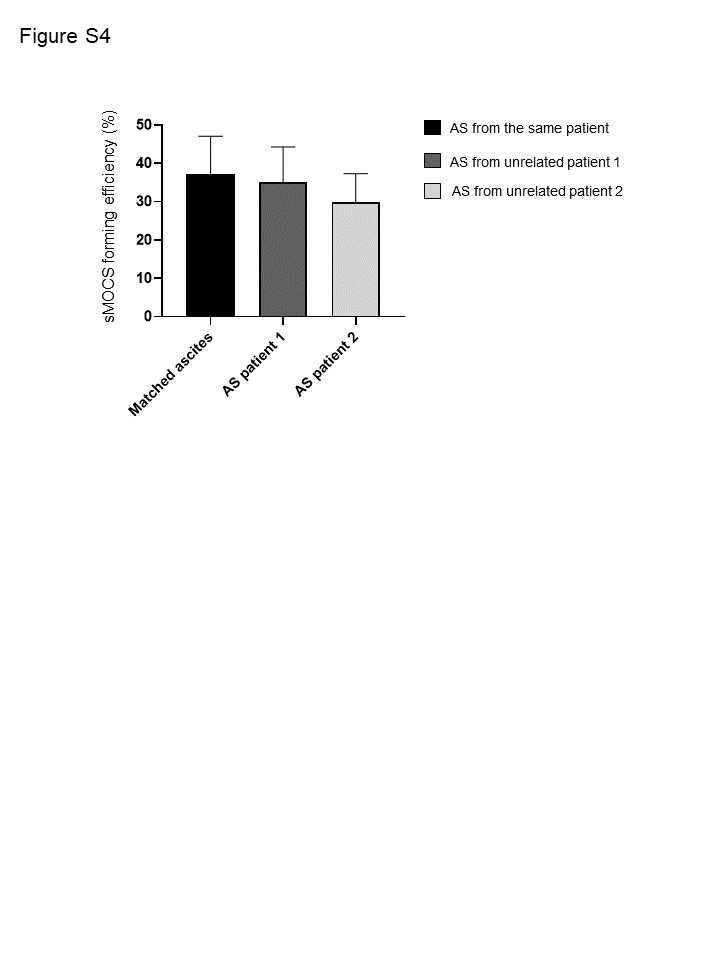

Supplement: Supplementary file 4 — Figure S4 [file 41418_2021_878_MOESM4_ESM.png]

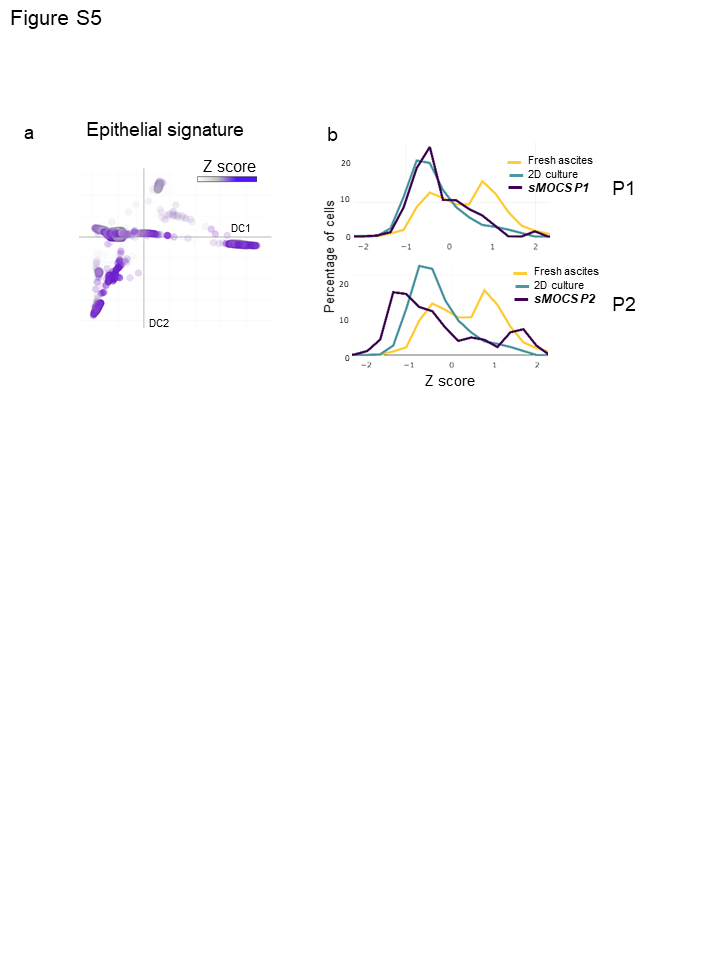

Supplement: Supplementary file 5 — Figure S5 [file 41418_2021_878_MOESM5_ESM.png]

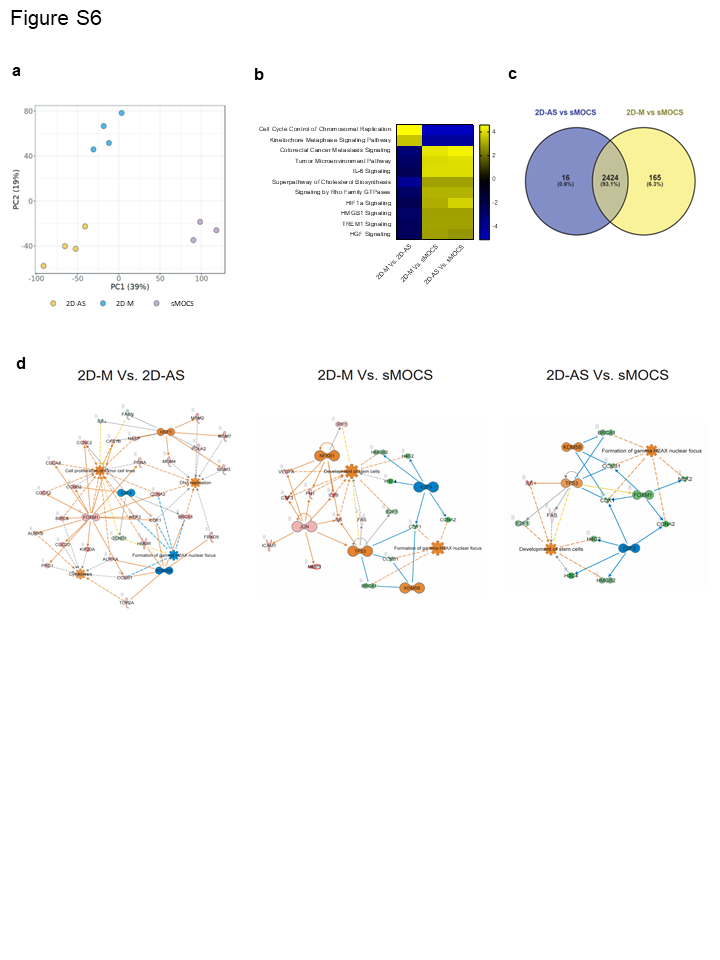

Supplement: Supplementary file 6 — Figure S6 [file 41418_2021_878_MOESM6_ESM.png]

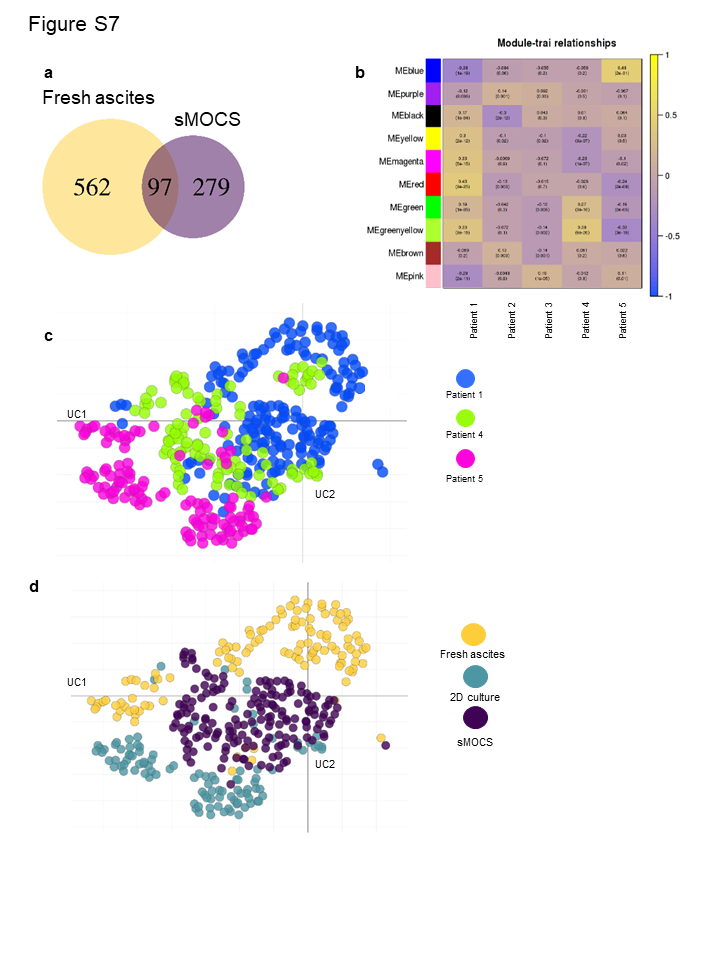

Supplement: Supplementary file 7 — Figure S7 [file 41418_2021_878_MOESM7_ESM.png]

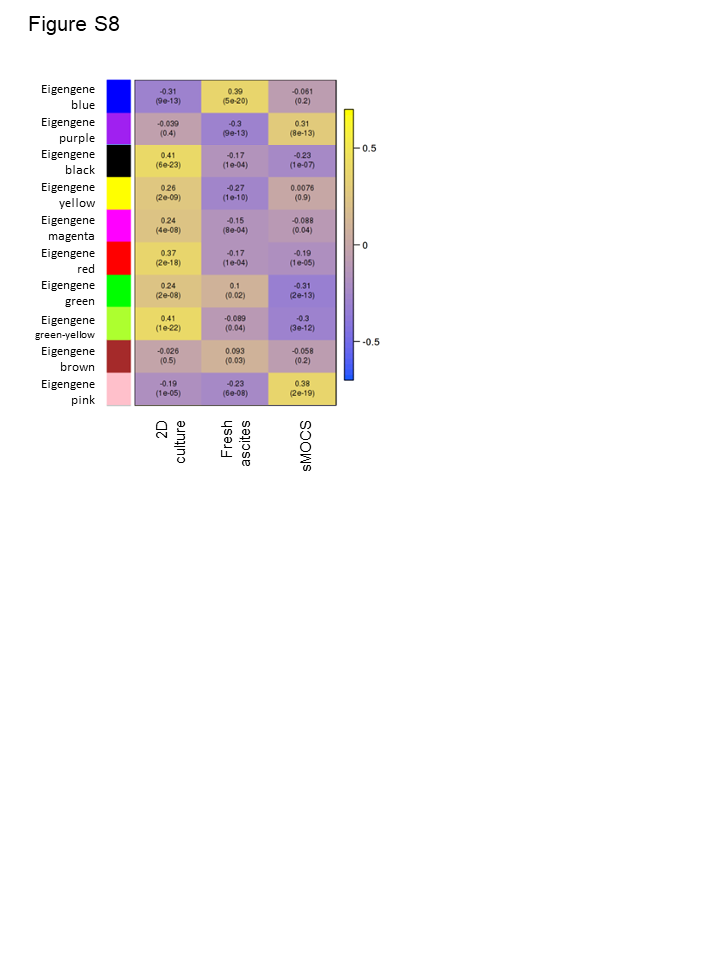

Supplement: Supplementary file 8 — Figure S8 [file 41418_2021_878_MOESM8_ESM.png]
